# Approaches to Control Crazing Deformation of PHA-Based Biopolymeric Blends

**DOI:** 10.3390/polym15214234

**Published:** 2023-10-26

**Authors:** Ramin Hosseinnezhad, Dhanumalayan Elumalai, Iurii Vozniak

**Affiliations:** Centre of Molecular and Macromolecular Studies, Polish Academy of Sciences, 90-363 Lodz, Poland; dhanumalayan.elumalai@cbmm.lodz.pl

**Keywords:** crazing, biopolymer, deformation, PHA, PBAT

## Abstract

The mechanical behavior of polymer materials is heavily influenced by a phenomenon known as crazing. Crazing is a precursor to damage and leads to the formation of cracks as it grows in both thickness and tip size. The current research employs an in situ SEM method to investigate the initiation and progression of crazing in all-biopolymeric blends based on Polyhydroxyalkanoates (PHAs). To this end, two chemically different grades of PHA, namely poly(hydroxybutyrate) (PHB) and poly(3-hydroxybutyrate-co-3-hydroxyvalerate) (PHBHV), were melt-blended with polybutyrate adipate terephthalate (PBAT). The obtained morphologies of blends, the droplet/fibrillar matrix, were highly influenced by the plasticity of the matrices as well as the content of the minor phase. Increasing the concentration of PBAT from 15 to 30 wt.% resulted in the brittle to ductile transition. It changed the mechanism of plastic deformation from single craze-cracking to homogeneous and heterogeneous intensified crazing for PHB and PHBHV matrices, respectively. Homogeneous tensile crazes formed perpendicularly to the draw direction at the initial stages of deformation, transformed into shear crazes characterized by oblique edge propagation for the PHBHV/PBAT blend. Such angled crazes suggested that the displacement might be caused by shear localized deformation. The crazes’ strength and the time to failure increased with the minor phase fibers. These fibers, aligned with the tensile direction and spanning the width of the crazes, were in the order of a few micrometers in diameter depending on the concentration. The network of fibrillar PBAT provided additional integrity for larger plastic deformation values. This study elucidates the mechanism of crazing in PHA blends and provides strategies for controlling it.

## 1. Introduction

Polyhydroxyalkanoates (PHAs) are a family of biodegradable and biocompatible polymers with diverse applications in various fields. Among them, polyhydroxybutyrate (PHB), polyhydroxybutyrate-co-hydroxyvalerate (PHBV), and poly(4-hydroxybutyrate) (P4HB) are the most commonly studied biopolymers [1,2]. Despite the significant advantages of PHAs, the widespread adoption of them in various industries has been hindered by certain drawbacks, e.g., low thermal and mechanical properties. To address these limitations while maintaining biodegradability, they are often blended with other biodegradable polymers, such as polylactide (PLA) and polycaprolactone (PCL). However, the inadequate compatibility and poor interfacial adhesion of the pairs result in phase separation, heterogeneous structures, and lower-than-expected mechanical performance [3,4,5]. In particular, PHAs can be mixed together with co-polyesters like poly butylene-co-succinate-co-adipate (PBSA) and polybutyrate adipate terephthalate (PBAT) at high temperatures and then cooled to create a solid blend that combines the desirable properties of both. The compatibility of these pairs is influenced by the ratio of compounds, the chemical structure of the polymers, and the processing conditions used to create the blend [6,7]. Zytner et al. conducted a successful analysis of PHBV/PBAT blends, reporting that the transition in morphology and mechanical properties occurred between 30% and 50% of PBAT loading. Increasing the PBAT content reduced the crystallinity of PHBHV, which leads to enhanced toughness and modified stiffness with limited interaction between the PHA and PBAT. Yixin Deng and colleagues demonstrated a substantial increase in elongation-to-break for PLA blends (from 10% to 300%) within the 10–20 wt.% PBAT composition range. At 20 wt.% PBAT, a validated co-continuous phase structure emerged, highlighting the potential to control interpenetrating network structures and achieve unique properties [8]. Despite the improvements, the structure–property relationship, approaches to control the morphology, and deformation mechanisms of the blend remain poorly understood [9,10,11].

In general, deformation at the molecular level is a combination of elastic, viscoelastic, and plastic consequent mechanisms: reversible stretching of bonds, segmental motions, and non-reversible slipping of molecular chains. While instantaneous elastic deformations are observed in most mechanical situations, anelastic and plastic deformations occur at higher strain levels and can be characterized by two micro-mechanisms, crazing and shear banding [12]. Crazes, visible as stress-whitened regions in pure PHA samples, form along a plane perpendicular to the major tensile stress and consist of densely packed fibrils separated by voids. These fibrils provide load-bearing capabilities and prevent craze coalescence. Factors such as stress levels, temperature, and material defects influence craze nucleation and the formation of microcracks. Under constant loading, craze growth follows the meniscus instability, driven by differences in hydrostatic pressure. Shear yielding at higher strains hints at the initiation of plastic deformations, preventing brittle fracture [13]. Tanaka et al. investigated the deformation behavior of poly([R]-3-hydroxybutyrate) (P(3HB)) and poly(4-hydroxybutyrate) (P(4HB)) under tensile stress using molecular simulation techniques. They found that the stretching deformation of P(3HB) occurred in a two-step process, involving the disentanglement of the helical structure and subsequent elongation of the molecule. On the other hand, the stretching deformation of P(4HB) followed a one-step mechanism similar to the second step of P(3HB) [14]. Contrary to poly(3HB) homopolymers, copolymers with 3-hydroxyvalerate (3HV) units show varied deformation structures, indicating increased flexibility and stretchability. However, the presence of 4-hydroxybutyrate (4HB) units limits the P(3HB) copolymer’s stretchability and changes the fracture morphology to a mushroom-type deformation, which is typically observed in PHAs with medium-chain-length monomers. This indicates that P(4HB) shares similar structural characteristics in terms of fracture behavior with these PHAs. [15,16]. Bejagam et al. employed molecular dynamics simulations to encompass the impact of PHAs’ chemical structure, involving side chain length, backbone length, and side chain functional groups. The findings indicated a decrease in Young’s modulus and yield stress as the carbon atoms increased in both the side chain and backbone. Moreover, the mechanical properties were significantly influenced by the functional groups, which affected interchain interactions [17]. 

Recent advances in microscopy techniques have enabled the observation and analysis of deformation, craze formation, and breakdown at the nanoscale. For instance, in situ scanning electron microscopy (SEM) has shown that the initiation and growth rates of crazing in PLA-based composites are influenced by the morphology and concentration of the minor phase, as well as strain rate [18]. Experiments have provided high-resolution images of the deformation and fracture processes to register the change of plastic deformation from the heterogeneous to homogeneous intensive crazing [19]. In another study, Hobbs and Barham employed optical and electron microscopic techniques to analyze the craze morphology of PHB under different conditions, including fresh, aged, and annealed states. They found that PHB undergoes crazing before failure regardless of the annealing history. After high-temperature annealing, a distinct craze morphology was observed, characterized by the formation of a dense zone of micro-crazes in thin films and a notable change in the fracture surface of bulk samples [20]. Despite the considerable research conducted to reinforce PHAs and to grasp the deformation mechanisms of polymers, current understanding of this field remains limited, highlighting a significant knowledge gap. In this study, we aim to investigate the specific features of crazing in PHAs which differ in chemical structure, crystallinity, and their interaction with the PBAT minor phase. The implementation of this work provides approaches for controlling the crazing mechanisms of PHA-based blends.

## 2. Experimental

### 2.1. Materials and Sample Preparation

Commercial grade PHAs, namely poly(hydroxybutyrate) (PHB) and poly(3-hydroxybutyrate-*co*-3-hydroxyvalerate) (PHBHV), with 12% 3-hydroxyvalerate (HV) units and a molar mass of 90,000 g mol^−1^, were purchased from Goodfellow and used as the matrices. Poly(butylene adipate-co-terephthalate) (PBAT) with the trade name ecoflex^®^ F Blend C1200 by BASF AG (Ludwigshafen am Rhein, Germany) was purchased and used to reinforce the PHAs. 15, 30, and 45 wt.% of PBAT were blended with PHB and PHBHV (all dried for 8 h at 60 °C) following the procedure reported previously [18]. Melt blends containing 15, 30, and 45 wt.% of PBAT were prepared using a co-rotating twin-screw extruder 2 × 20/40D EHP (Zamak Mercator, Skawina, Poland) operating at 120 rpm. The temperature zones in the extruder barrel were maintained at 155 °C, 160 °C, 160 °C, 165 °C, 170 °C, 170 °C, 170 °C, 170 °C, 165 °C, and 155 °C. Additional blending was followed by the extrusion of pelletized extrudates using a single-screw extruder (PlastiCorder PLV 151, Brabender; D = 19.5 mm, L/D = 25, and 20 rpm) equipped with the 12 mm wide, 0.8 mm thick, and 100 mm long slit die. Temperatures in the zones were 175 °C, 150 °C, and 145 °C from the feed section to the die, respectively. The slit die process parameters were as follows: temperature 135 °C and pressure 65.0 MPa. The extrudates in the form of tapes, approximately 0.5 mm thick and 10 mm wide, were transported on a conveyor belt at T = 25 °C.

### 2.2. Mechanical and Thermal Properties

Tensile properties of PHAs, PBAT, and blends were measured in Instron-5582 (Universal Testing Machine, High Wycombe, UK) at a strain rate of 5%/min according to ISO 527-2 [21]. For the examination of the mechanical properties, specimens of the gauge length of 25 mm and a width of 5 mm (ISO 527-2, type 1BA) were cut out from compression-molded samples for pure polymers and from extruded tapes along the extrusion directions for blends. The impact strength was determined in Resil-5.5 (Instrumented Impact Tester, CEAST, Italy) as per ISO 8256 [22]. Specimens with a gauge length of 25 mm and width of 3 mm were struck by a hammer at a speed of 2.9 m/s and energy of 1 J.

The melting and non-isothermal crystallization of samples were examined by employing an indium-calibrated differential scanning calorimeter DSC Q20 (TA Instruments, New Castle, DE, USA). Specimens weighing 6–10 mg were heated to 180 °C, annealed for 3 min, and cooled down to crystallize, at a constant heating/cooling rate of 10 °C min^−1^. The entire thermal treatment was performed under nitrogen flow.

### 2.3. Scanning Electronic Microscopy (SEM)

Prior to investigating the morphology of cryogenic fractures with JEOL JSM-5500 LV (Scanning Electron Microscope, Tokyo, Japan), PHAs were gently etched using formic acid at room temperature to dissolve PHB and PHBHV fraction [11]. The surfaces of the samples were coated with gold. Gatan MT200 (Microtest Tensile Stage, Suffolk, UK), connected to a microscope, facilitated in situ observations of the tensile test according to ASTM D638 [23]. Specimens were coated with carbon and deformed at 0.2 mm/min rate following a procedure described previously [18]. 

## 3. Results and Discussion

### 3.1. Morphological Development

**Figure 1:**
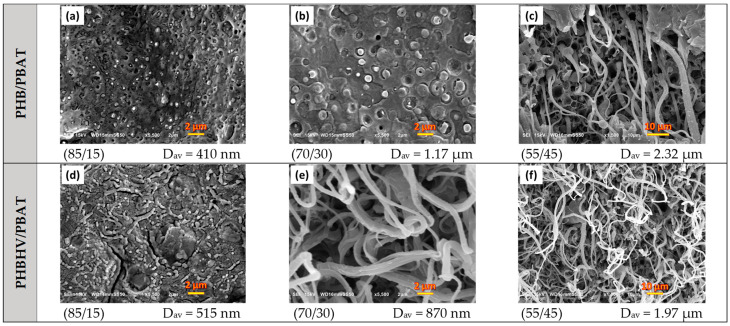
SEM images revealing the cryofractured surfaces of PHB/PBAT (**a**–**c**) and PHBHV/PBAT (**d**–**f**) samples and their respective compositions of 85/15, 70/30, and 55/45 wt.%.

Figure 1 shows the microstructures of blends, where all of the samples reveal a typical two-phase structure with micro/nanosized PBAT droplets and fibers dispersed in the matrices. However, the morphology of the samples is influenced by the chemical structures of employed PHAs as they interact differently with the dispersed PBAT. The formation of PBAT micro/nanodroplets in PHB/PHBAT (85/15) and PHB/PHBAT (70/30) blends indicates that an intensive shear rate is realized in the course of blending, resulting in the large deformation of the PBAT phase and the minimized agglomeration of the shear-split dispersed phase. The size of the PBAT droplets increases with increasing content of the PBAT copolyester. In particular, the average particle size increases from 0.410 to 1.170 µm for the blends of PHB with 15 and 30 wt.% of PBAT, respectively. In contrast, the morphology of PHBHV/PBAT (85/15) suggests that the viscous force of the matrix is insufficient to overcome the cohesive strength of PBAT threads. The presence of the hydroxyvalerate units in PHBHV introduces structural similarities to PBAT, such as the presence of ester groups. These similarities increase flexibility, reduce crystallinity, and facilitate intermolecular interactions, resulting in better compatibility. 

SEM micrographs of PHB/PBAT (55/45), PHBHV/PBAT (70/30), and (55/45) reveal different morphologies, i.e., fibril/matrix. Long PBAT fibers are generated with large aspect ratios. The fibers are 2.32, 0.87, and 1.97 µm in diameter in PHB/PBAT (55/45), PHBHV/PBAT (70/30), and PHBHV/PBAT (55/45), respectively. Prior to the entry of the extruder, the dispersed phase in the extruded melt is present predominantly in the form of micro-droplets. Smaller droplets can collide and coalesce into larger droplets, which are transformed into fibrils. The fibrils are formed within the area between the extruder screw and the extruder walls, where they are entangled. Entanglement occurs during compounding and not during extensional flow in the slot capillary. The slot capillary serves to dampen the flow and increase the residence time of the mixture in the extruder. 

### 3.2. Mechanical Properties

**Figure 2:**
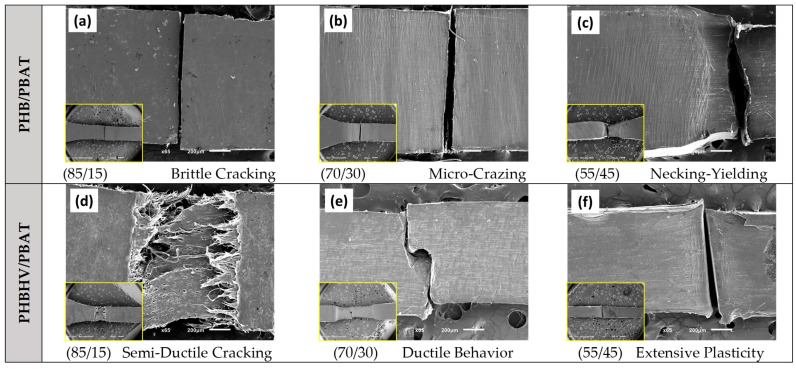
SEM images of PHB/PBAT (**a**–**c**) and PHBHV/PBAT (**d**–**f**) samples with different compositions upon tensile deformation.

The SEM images presented in Figure 2 depict the tensile deformation behavior of the blends with different compositions. In the case of PHB/PBAT (85/15), a sharp brittle fracture is observed, indicating a single craze to crack and subsequent failure. The absence of significant crazing in this sample with no apparent plastic deformation before reaching its breaking point, implies that the material exhibits a limited ability to absorb energy. Upon increasing the PBAT concentration to 30 wt.%, a substantial number of micro crazes prior to failure are visible as the whitening of the material around the fracture zone. The presence of these crazes indicates the improved distribution of stress over the sample, which facilitates reaching higher strain values. Further increasing the PBAT concentration to 45 wt.%, the sample demonstrates even higher ductility and exhibits fully localized necking and partial plastic deformation before eventual breakage.

In contrast, PHBHV/PBAT (85/15) shows a semi-plastic fracture at the yielding point, where the craze-to-crack development is accompanied by localized partial plastic flow at the fracture surface. The PHBHV/PBAT (70/30) and (55/45) compositions are capable of sustaining extensive plastic deformation over an extended region after necking and yielding; however, they eventually break, typically from the middle of the extended and plastically deformed regions. The observed differences in deformation modes, including brittle fracture, micro crazing, necking, and plastic deformation, indicate the influence of PBAT concentration and morphology over the obtained mechanical properties. 

**Figure 3:**
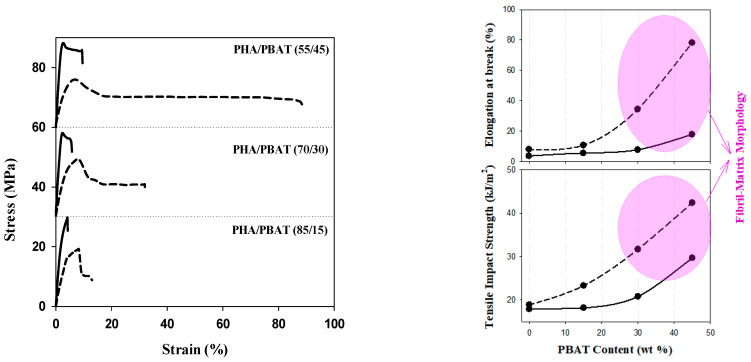
Stress–strain curve as well as elongation at break and tensile impact strength for PHB/PBAT (solid line) and PHBHV/PBAT (dashed line). To enhance clarity, the stress–strain curves have been shifted along the stress axis 30 MPa and 60 MPa in the 70/30 and 55/45 compositions, respectively.

Figure 3 illustrates the stress–strain relationships, as well as elongation at break and tensile impact strength, highlighting the significance of PBAT content over the morphology, strength, and plasticity of the blends. As PBAT is introduced to PHB and its content increases to 45 wt.%, elongation at break reaches around 20%. This is accompanied by a 50% increase in the tensile impact strength and a slight reduction in Young’s modulus from 3.0 GPa for pure PHB to 2.71 GPa for PHB/PBAT (55/45). These results align with the microstructure observations, indicating a significant enhancement in the plasticity of the blend to undergo necking and ductile yielding. The incorporation of the hydroxyvalerate unit decreases Young’s modulus from 3.0 GPa for PHB to 1.9 GPa for PHBHV. The elongation at break is still below 10% for PHBV/PBAT (85/15) and Young’s modulus is 1.67 GPa. However, the elongation at break and tensile impact strength of the blends increased with higher PBAT content, and the highest reinforcing effect was achieved for PHBHV/PBAT (55/45) with an 800% increase in strain at break and a 200% increase at tensile impact strength. This is attributed to the formation of a physically entangled network of PBAT fibers. Blends with a fibril matrix morphology possess a larger specific surface area and stronger interphase interactions in comparison to those with droplet matrix morphology.

### 3.3. Micro-Mechanisms of Deformation

#### 3.3.1. Brittle/Semi-Ductile Cracking

**Figure 4:**
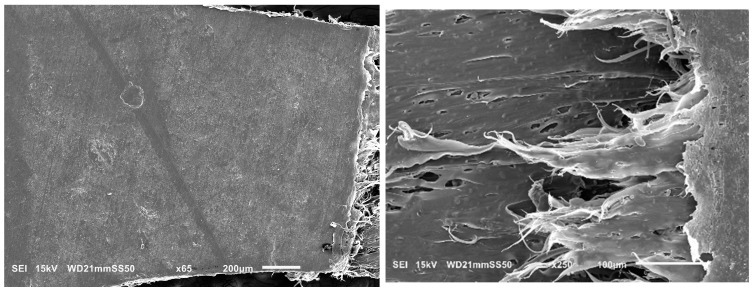
SEM micrographs of PHBHV/PBAT (85/15) blend during tensile testing, showing the craze-crack breakdown without considerable crazing adjacent to the crack (**left**) and partially localized plasticity at the crack zone (**right**).

Further investigation into the deformation mechanism of blends was carried out by conducting in situ tensile testing in the SEM. This analysis aimed to elucidate the factors contributing to the transition from brittle to ductile behavior in the blends, as well as to clarify the influence of obtained morphologies on the deformation mechanism. Previous discussions of mechanical improvements highlighted that PHB/PBAT (85/15) exhibits a rapid propagation of a single craze to crack, leading to brittle fracture within the apparent elastic region, below the macroscopic yield stress. This single-craze development, leading to cracking and the subsequent fracture, occurs perpendicularly to the draw direction and in the absence of any neighboring crazes (Figure 4, left). 

However, partial plasticity is observed in a narrow zone adjacent to the fracture surface for PHBHV/PBAT (85/15). This semi-ductile cracking occurs due to the localized development of plastic deformation in the neighborhood of the fracture, where the sample experiences faster growth in plastic strain compared to the overall bulk sample (Figure 4-right).

#### 3.3.2. Intensive Homogenous Crazing

**Figure 5:**
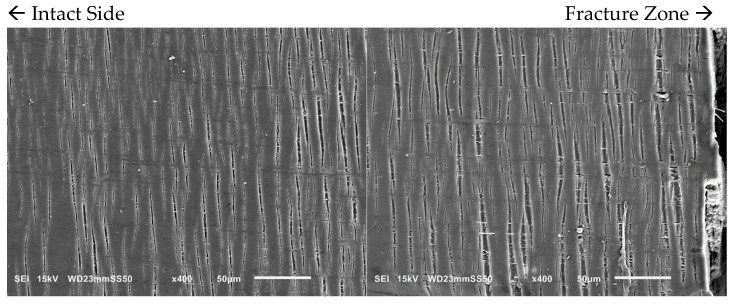
SEM micrographs of PHB/PBAT (70/30), revealing intensive homogeneous tensile crazing during in situ tensile.

With an increase in PBAT content from 15 to 30 wt.%, the number of crazes becomes more pronounced as observed through in situ SEM micrographs (Figure 5). The density of these crazes is higher, and they are well distributed throughout the sample. Such extensive crazing and the consequent load transmission prevent the simple propagation of a single craze into a crack, allowing the yielding and enhancement of the ductility of the samples prior to fracture. In the PHB/PBAT (70/30) blend, the tensile crazes are homogeneously dispersed over the sample. However, the width and length of these crazes gradually increase from the intact side toward the fracture zone. On the intact side, crazes with a length of 15–30 μm propagate perpendicularly to the applied tensile stress. The primary cause of crazing growth on the intact side is the forward expansion of the crazing tip, with a minimal increase in thickness. The width of these tensile crazes ranges from 1–3 μm, accompanied by some derived sub-crazing. On the other side close to the fracture zone, crazes still continue to grow perpendicular to the tensile stress direction reaching lengths of 55–90 μm. The thickness increases significantly up to 13 μm, eventually leading to continuous thickening and fracture as the crazing tip extends forward.

#### 3.3.3. Intensive Heterogeneous Crazing

**Figure 6:**
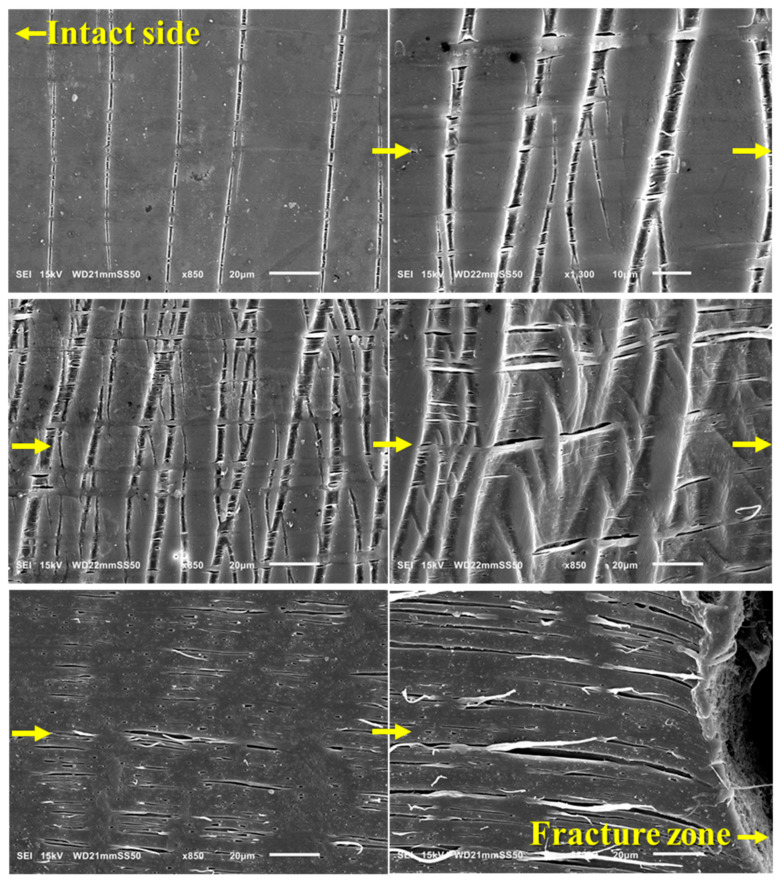
SEM micrographs of PHBHV/PBAT (70/30), revealing intensive heterogeneous crazing during in situ tensile. The sequence corresponds to the captured images from the intact side of the sample, moving gradually to the fracture zone, as indicated by the direction of the arrows.

In contrast to the homogeneous crazing deformation in PHB/PBAT (70/30) with a droplet matrix morphology, significant changes in the plastic deformation mechanism are observed in the PHBHV matrix when 30 wt.% of PBAT is added. These changes are evident throughout the whole sample: from the long tensile crazes on the intact side, with a significant length ranging from 100–250 μm, while the thickness remains around 3 μm, to the evolution of shear crazes in between, and finally, plastic flow as the fracture zone is approached (Figure 6). 

The direction of tensile crazes on the intact side remains regularly perpendicular to the applied tensile stress, and the crazing tip continues to expand forward. Compared to the PHB/PBAT (70/30), the forward expansion rate of the crazing tip is higher. 

**Figure 7:**
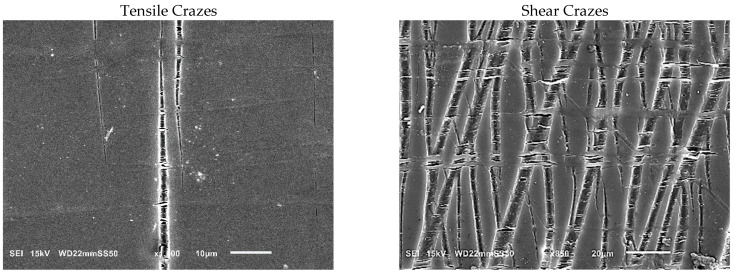
SEM micrographs of tensile and shear crazes for PHBHV/PBAT (70/30).

As we progress from the intact side toward the fracture zone, a notable evolution in crazing is observed. Initially, the crazes become thicker, reaching up to 15 μm, and adopt a distinctive structure consisting of PBAT fibers interspersed with nucleated adjacent voids. These fibers, aligned perpendicularly to the interface of the craze bulk, facilitate load distribution and effectively stabilize the growth of crazes by spanning the surfaces and preventing the rapid propagation of cracks. The voids next to fibers have a thickness ranging from 0.8 to 1.4 μm and a length of around 5–10 μm. As plastic deformation progresses, they coalesce with each other to form long extended voids that are aligned with the principal tensile stress direction and that have reached a length of up to 40 μm. PHB/PBAT (70/30) shows heterogenous crazing where the perpendicular tensile crazes transform into shear crazes, which emerge at an angle of approximately 30–35 degrees relative to the specimen’s short axis, suggesting an isotropic state achieved through a well-dispersed network of physically entangled PBAT fibers (Figure 7) [24]. 

As we approach the fracture zone, the crazes flow and diffuse into the plastically deformed region, characterized by substantial permanent strain. In this plastically oriented zone, such a yielding process greatly enhances the ductile behavior and imparts a high level of toughness to the sample.

#### 3.3.4. Yield of Crazes

**Figure 8:**
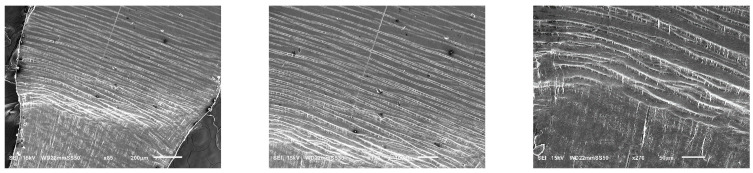
SEM micrographs (different magnifications) of the PHB/PBAT (55/45) sample during tensile testing showing regular formation, development, and yield of parallel tensile crazes.

In the case of the blends containing 45 wt.% PBAT, the predominant mechanism driving plastic deformation is the extensive formation of crazes and their yielding into the plastic flow zone. Figure 8 illustrates the structural evolution of PHB/PBAT (55/45) during a tensile test showing that on the intact side of the blend, numerous straight tensile crazes are regularly aligned next to each other, all propagated perpendicular to the tensile direction. These parallel tensile crazes reach a length ranging from 200 to 550 μm, with a thickness of approximately 5 μm. Approaching the plastic flow zone, they become thicker, up to 20 μm, and finally detach and flow to the plastic zone with the same parallel regularity. 

**Figure 9:**
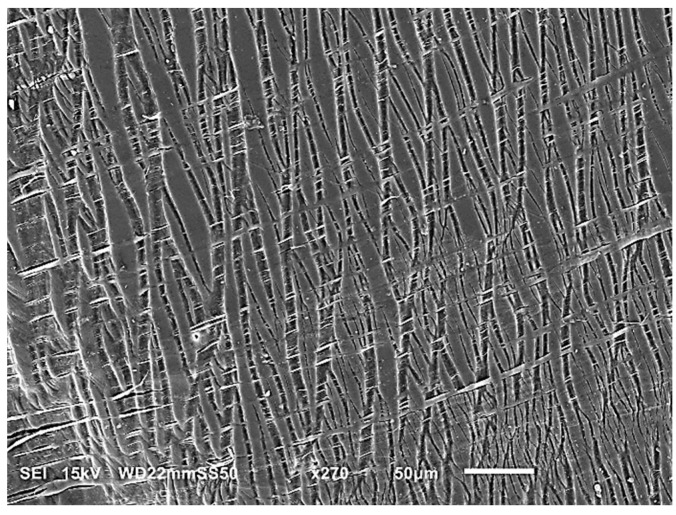
SEM micrograph of PHBHV/PBAT (55/45) during tensile testing showing yield of angled shear crazes.

In contrast to the tensile crazes of PHB/PBAT (55/45), the growth direction of crazes for PHBHV/PBAT (55/45) deviates from being perpendicular to the direction of tensile stress. Additionally, a significant number of micro sub-crazes, with thicknesses in the range of a few micrometers, emerge along the interior of the main crazes without any discernible regularity. Unlike the continuous growth from the top to the bottom edge of the sample, these sub-crazes exhibit relatively shorter lengths, typically ranging from 10 to 30 μm. The SEM image in Figure 9 provides further insight into the angled shear crazes and the subsequent yield of these crazes into the plastic zone, revealing the presence of long PBAT fibers interspersed between the main crazes. These cross-tie fibers contribute to the limited lateral load-bearing capacity of the shear crazes.

### 3.4. Thermal Properties 

**Figure 10:**
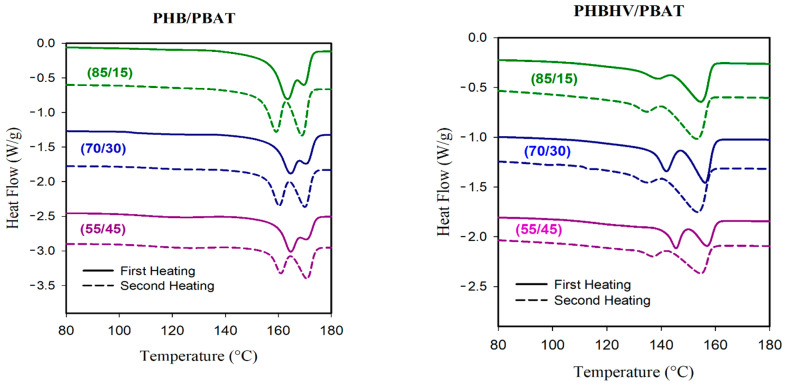
DSC heating curves suggest melt/recrystallization and co-crystallization processes for PHB/PBAT and PHBHV/PBAT blends, respectively. The solid and dashed lines correspond to the first and second heating scans.

The thermal properties of PHB/PBAT and PHBHV/PBAT blends were investigated through DSC analysis with respective heating and cooling scans. PBAT has a shallow T_m_ transition peak at ~121 °C; however, this transition is barely visible for PHB/PBAT (85/15) and PHB/PBAT (70/30), indicating that the majority of crystals belong to PHB. However, the heating cycles of PHB/PBAT (55/45) sample, with fibrillar matrix morphology, clearly exhibit the characteristic signal of PBAT (Figure 10). 

The first heating scan of all PHB/PBAT samples also shows a prominent T_m_ transition around 164 °C and a shoulder peak of around 170 °C. DSC results at a slow heating rate of 1 °C min^−1^ prove that the relative areas of the peaks change by lowering the heating rate, suggesting that these head–shoulder peaks can be attributed to a melt/recrystallization process. The head (lower temperature peak) corresponded to the melting of the crystals formed at the crystallization temperature while the shoulder peak was caused by the crystals rearranging during heating. In the second heating scan, the transition around 164 °C starts to depreciate, and the peak around 170 °C becomes prominent.

PHBHV/PBAT heating curves show two distinct melting peaks rather than head–shoulder peaks. The DSC conducted at a slower heating rate of 1 °C min^−1^ suggested that PBAT may partially co-crystallize with PHBHV to create a lower melting peak. The following mechanism may elucidate the formation of the cocrystals that melt at the first peak. Upon the cooling of the molten PHBHV/PBAT blend, PHBHV chains are presumably ready to crystallize before the PBAT chains. However, due to the sluggish phase segregation between PHBHV and PBAT, adjacent PBAT chains could be trapped by PHBHV chains and be pulled into the crystalline phase. In PHBHV/PBAT blends with fibrillar matrix morphology and improved compatibility, the PBAT chains with higher concentrations of 30 and 45 wt.% are more susceptible to being dragged into the growing front of PHBHV crystals and, as a result, the height of the relevant peak is increased.

**Figure 11:**
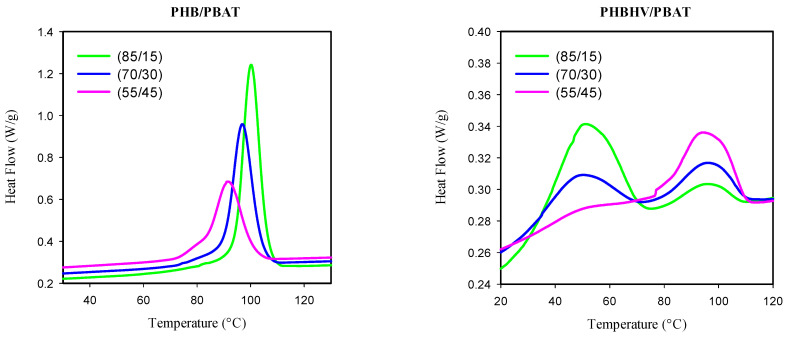
DSC cooling exotherms of PHB/PBAT and PHBHV/PBAT blends.

The mechanism of crystal formation was further investigated by analyzing the cooling exotherms (Figure 11). The exotherms of PHB/PBAT show a single crystallization peak, which shifts to the lower temperature regime with increasing PBAT content and finally, at 45 wt.% of PBAT, obtains a shoulder, hinting at the small population of PBAT crystals being formed. On the other hand, PHBHV/PBAT exhibited two exothermic peaks of T_c_ in which one falls in the low-temperature zone (~50 °C). The height of the low-temperature peak retards with the addition of PBAT into the PHBHV matrix. On the other hand, the height of the high-temperature peak (~95 °C) is intensified with the increase in PBAT content. indicating a higher contribution of PBAT chains in the co-crystallization process with PHBHV. These results are in agreement with the obtained morphologies and mechanical properties: they enhance the likelihood of preserving the in situ-generated fibers of PBAT before they potentially break up into micro- or nanodroplets. 

## 4. Conclusions

Several key factors, including the concentration of the minor phase, the morphology (droplets versus fibers), and the ability of fibers to form a network of physical entanglements, are found to contribute to the crazing deformation of PHB/PBAT and PHBHV/PBAT, although the mechanisms are somewhat different in the two blends. 

The introduction of PBAT into the PHB matrix, particularly with an increased concentration, exerted a notable influence on the mechanical behavior and deformation mechanisms of the resulting blends. A compelling observation was the extensive generation of parallel tensile crazes within the plastic deformation region. These crazes exhibited a striking characteristic of homogeneous alignment perpendicular to the applied tensile test direction. As the PBAT content was augmented, the prevalence and alignment of these tensile crazes became more pronounced and significantly impacted the macroscopic yielding behavior of the blend and the overall mechanical properties and ultimate failure characteristics.

The hydroxyvalerate units in PHBHV enhanced the interaction of PHBHV and PBAT. At 15 wt.% of PBAT, characterized by droplet-like morphology, the fracture occurs through semi-ductile cracking. Adding 30 wt.% of PBAT with fibrillar morphology induces high plasticity, altering the deformation mechanism to heterogenous crazing where the tensile crazes on the intact side of the sample develop into shear crazes, which emerge at an angle of approximately 30–35 degrees relative to the specimen’s short axis. The PBAT fibers contribute to the spanning of the crazes’ surfaces until they yield. Microvoids generated next to fibers within the tensile crazes merge with the other microvoids of the neighboring crazes. As a result, new long crazes are formed and propagate along the principal tensile direction in the plastically deformed region. As the PBAT content of this blend increased, and the in situ-generated fibers showed an elevated inclination to be solidified through co-crystallization with PHBHV. Thus, a physically entangled network of PBAT fibers for PHBHV/PBAT (55/45) was formed, featuring the most improvements in mechanical properties.

## Data Availability

Not applicable.
